# SRC family kinase (SFK) inhibition reduces rhabdomyosarcoma cell growth *in vitro* and *in vivo* and triggers p38 MAP kinase-mediated differentiation

**DOI:** 10.18632/oncotarget.3043

**Published:** 2015-02-03

**Authors:** Nadia Casini, Iris Maria Forte, Gianmarco Mastrogiovanni, Francesca Pentimalli, Adriano Angelucci, Claudio Festuccia, Valentina Tomei, Elisa Ceccherini, Domenico Di Marzo, Silvia Schenone, Maurizio Botta, Antonio Giordano, Paola Indovina

**Affiliations:** ^1^ Department of Medicine, Surgery and Neuroscience, University of Siena and Istituto Toscano Tumori (ITT), Siena, Italy; ^2^ Oncology Research Center of Mercogliano (CROM), Istituto Nazionale per lo Studio e la Cura dei Tumori “Fondazione Giovanni Pascale”, IRCCS, Naples, Italy; ^3^ Department of Biotechnological and Applied Clinical Sciences, University of L'Aquila, L'Aquila, Italy; ^4^ Pharmacy Department, University of Genoa, Genoa, Italy; ^5^ Department of Biotechnology, Chemistry and Pharmacy, University of Siena, Siena, Italy; ^6^ Sbarro Institute for Cancer Research and Molecular Medicine, Center for Biotechnology, College of Science and Technology, Temple University, Philadelphia PA, USA

**Keywords:** rhabdomyosarcoma, SRC family inhibition, NOTCH3, muscle differentiation, p38 MAPK, YES

## Abstract

Recent data suggest that SRC family kinases (SFKs) could represent potential therapeutic targets for rhabdomyosarcoma (RMS), the most common soft-tissue sarcoma in children. Here, we assessed the effect of a recently developed selective SFK inhibitor (a pyrazolo[3,4-*d*]pyrimidine derivative, called SI221) on RMS cell lines. SI221, which showed to be mainly effective against the SFK member YES, significantly reduced cell viability and induced apoptosis, without affecting non-tumor cells, such as primary human skin fibroblasts and differentiated C2C12 cells. Moreover, SI221 decreased *in vitro* cell migration and invasion and reduced tumor growth in a RMS xenograft model. SFK inhibition also induced muscle differentiation in RMS cells by affecting the NOTCH3 receptor-p38 mitogen-activated protein kinase (MAPK) axis, which regulates the balance between proliferation and differentiation. Overall, our findings suggest that SFK inhibition, besides reducing RMS cell growth and invasive potential, could also represent a differentiation therapeutic strategy for RMS.

## INTRODUCTION

Rhabdomyosarcoma (RMS) is the most common soft-tissue sarcoma in children. This highly malignant tumor has traditionally been considered to arise from committed skeletal muscle precursor cells that fail to differentiate [1], although some RMSs develop in organs lacking a skeletal muscle component [2, 3].

RMS is classified into two major subtypes: embryonal (ERMS) and alveolar (ARMS) RMS, the first being the most common type, accounting for approximately two thirds of cases [1, 3]. ERMS most commonly occurs in the head and neck region and genito-urinary tract of younger children (1–14 years), whereas ARMS occurs more frequently in the extremities and trunk region of older children and adolescents [1, 3].

Over the last decades, significant progresses have been made in RMS therapy through the use of chemotherapy in association with surgery and radiation therapy as adjunct treatments. Indeed, in patients with low-grade, localized ERMS, the five-year survival rate is > 85% [3]. Nevertheless, this rate decreases with increased age and tumor stage. ARMS is more aggressive than ERMS, showing a poorer response to therapy and a worse prognosis, with a five-year survival rate of approximately 50%, which dramatically decreases to 10–30% for metastatic ARMS [4]. Moreover, since RMS primarily affects children and adolescents, there are concerns about long-term adverse effects of the treatment, which might cause growth and developmental problems and increase the risk of secondary cancers [3].

Therefore, the development of more selective targeted therapies against RMS is an urgent need. Understanding the molecular mechanisms leading to RMS onset and progression is essential to identify rational therapeutic strategies. RMS is thought to arise as a consequence of multiple defects leading to a disrupted balance between the mutually exclusive and strictly coordinated proliferation and myogenic differentiation processes [1, 3]. The most common alteration detected in ERMS is loss of heterozygosity on the short arm of chromosome 11 (11p15.5), whereas ARMS is characterized by the t(2;13)(q35;q14) translocation in approximately 70% of cases and by the t(1;13)(p36;q14) translocation in approximately 10% of cases, which generate PAX3-FKHR and PAX7-FKHR fusion proteins, respectively [2, 3]. However, many other molecular alterations in cell cycle and differentiation pathways have been identified [1, 3–5].

Hyperactivation of the SRC family kinases (SFKs) has been observed in many human cancers. These kinases are considered attractive targets in cancer therapy because of their central role in controlling cell proliferation, apoptosis, angiogenesis and invasion [6]. Recent data show that SFKs are hyperactivated also in RMS cell lines [7, 8] and, consistently, SFK inhibition, mediated by the multitargeted tyrosine kinase inhibitor dasatinib, induces an antiproliferative effect on RMS cell lines, both *in vitro* and in xenotrasplanted mice [8], and a reduction in cell migration [7]. These observations suggest that SFKs could represent therapeutic targets also for RMS.

We recently synthesized new pyrazolo[3,4-*d*]pyrimidine derivative SFK inhibitors, binding the ATP pocket of SFKs, which proved to be good antiproliferative and proapoptotic agents in several tumor types [9–12]. Here, we focused on one of these new SFK inhibitors, the SI221 compound, which was able to reduce cell viability in both ERMS and ARMS cell lines, without significantly affecting non-tumor cells. SI221 also decreased RMS cell migration and invasion *in vitro* and reduced tumor growth in xenotrasplanted mice. Interestingly, a kinase activity screening assay showed that SI221 is mainly effective against the SFK member YES, which has recently been identified as an important player in RMS development [8]. Moreover, our data suggest that SFK inhibition through SI221 could rescue the differentiation program in RMS cells (evaluated by both myogenic marker expression and cell morphology) by inhibiting NOTCH3 receptor, which hinders muscle differentiation, and activating p38 mitogen-activated protein kinase (MAPK), which promotes differentiation.

## RESULTS

### Cytotoxic effect of pyrazolo[3,4-*d*]pyrimidine SFK inhibitors on RMS cell lines

**Figure 1:**
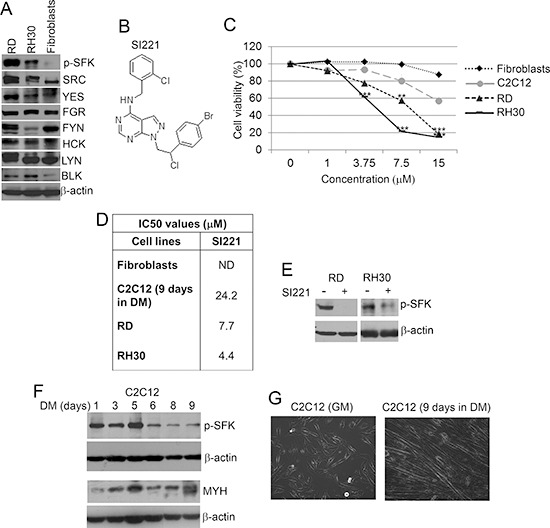
Effect of SI221 on cell viability of RMS and non-tumor cells **(A)** Representative western blots of the active form of SFKs, i.e. phospho-SFK (p-SFK), and total levels of SFK members (SRC, YES, FGR, FYN, HCK, LYN and BLK) in RD and RH30 cell lines and primary human skin fibroblasts. An anti-β-actin antibody was used for a loading control. **(B)** Structure of the pyrazolo[3,4-*d*]pyrimidine derivative SFK inhibitor SI221. **(C)** MTS analysis of cell viability of primary human skin fibroblasts, C2C12 cells grown in differentiation medium (DM) for 9 days and RMS cell lines (RD and RH30) 72 hours after treatment with SI221. Results are reported as means of three independent experiments, each conducted in triplicate, and expressed as percentages of cell viability calculated with respect to control cells treated with DMSO alone. The absorbance values of treated and control samples were subjected to one-way Anova with Dunnett post-test. Statistically significant differences between treated and control cells are indicated with **: very significant (*P* < 0.01) and ***: extremely significant (*P* < 0.001). **(D)** Table reporting the IC50 values of SI221 on non-tumor and RMS cells. SI221 was ineffective on fibroblasts at the concentrations used and, thus, the IC50 value was not determined (ND). **(E)** Representative western blots showing the decrease in phospho-SFKs in RD and RH30 cell lines treated with SI221 for 72 hours with respect to control cells treated with DMSO alone. An equal loading of proteins was verified with an anti-β-actin antibody. **(F)** Representative western blots showing the expression of phospho-SFKs and myosin heavy chain (MYH) in C2C12 cells grown in DM for 1–9 days. An anti-β-actin antibody was used for a loading control. **(G)** Representative micrographs showing the change in C2C12 morphology after 9 days in DM with respect to C2C12 cells grown in growth medium (GM). Original magnification: 10X.

Before testing our SFK inhibitors, we analyzed the expression of the active form of SFKs (phospho-SFKs) and total levels of SFK members (SRC, YES, FGR, FYN, HCK, LYN, BLK and LCK) in two RMS cell lines (RD and RH30, representing the ERMS and ARMS subtypes, respectively) and in non-neoplastic cells (primary human skin fibroblasts) for comparison. Both RMS cell lines expressed the active phosphorylated form of SFKs, which were almost undetectable in fibroblasts (Figure 1A). Total SRC, YES, FGR, FYN, HCK, LYN and BLK were all expressed in all the cell lines (Figure 1A), whereas LCK was not expressed (data not shown).

We then treated the RMS cell lines with a panel of new pyrazolo[3,4-*d*]pyrimidine SFK inhibitors at concentrations ranging from 1 to 15 μM for 72 hours and evaluated cell viability by MTS assay. To rule out possible cytotoxic effects of these molecules on non-neoplastic cells, our SFK inhibitors were also tested on fibroblasts and a mouse myoblast cell line, C2C12, which represent a well-established model to recapitulate skeletal muscle differentiation. C2C12 cells were grown in differentiation medium (DM) for 9 days to obtain cells mimicking the non-tumor muscle counterpart. We observed significant cytotoxic effects on RMS cell lines with all the molecules tested (data not shown). We focused in particular on one compound, SI221 (Figure 1B), which resulted effective on both RMS cell lines and showed low or no toxicity on differentiated C2C12 cells and fibroblasts, respectively, at the concentrations tested (Figure 1C).

To assay SI221 selectivity towards the various SFK members, we tested this small molecule against a panel of 29 different kinases, including 8 SFKs. The results demonstrated that SI221 specifically inhibits the kinase activity of SFK members, without significantly affecting the activity of the other kinases included in the panel (Supplementary Table 1). In particular, SI221 showed to be mainly effective against the SFK member YES, which seems to have an oncogenic role in both ERMS and ARMS [8].

We also compared by MTS the cytotoxic effect of our compound with that of the well-known SFK inhibitor PP2, which showed an efficacy lower than or similar to that of SI221 on RD and RH30 cells, with a half maximal inhibitory concentration (IC50) of 19.12 μM and 3.79 μM, respectively (Supplementary Figure 1).

To verify that SI221 inhibits SFK activation in RMS cell lines, we evaluated the expression of the active phosphorylated form of SFKs 72 hours after treatment with SI221 at its IC50 values (indicated in Figure 1D). As expected, the molecule caused a strong decrease in phospho-SFK level (Figure 1E).

The observation that SI221 was cytotoxic to RMS cells expressing the active form of SFKs and not to fibroblasts, in which active SFKs were almost undetectable, seems to confirm SI221 specificity towards active SFKs. To verify further that SI221 effectiveness is dependent on the activation status of SFKs, we analyzed the expression of the active form of SFKs also in C2C12 cells. We observed that SI221 cytotoxicity to C2C12 cells varied according to changes in the activation status of SFKs during differentiation. Indeed, consistent with a previous paper [13], SFK activation decreased during C2C12 differentiation (Figure 1F), which was evaluated by both myosin expression (Figure 1F) and cell morphology (Figure 1G), and SI221 effectiveness decreased accordingly. In particular, SI221 was effective on C2C12 cells still expressing high levels of the active form of SFKs after 5 days in DM (IC50: 4.5 μM), whereas it caused only a low toxicity on C2C12 cells grown in DM for 9 days and expressing a low level of active SFKs (IC50: 24.2 μM).

### SFK inhibition induces apoptosis in RMS cell lines

To evaluate whether the cytotoxic effect induced by SFK inhibition on RMS cells was due to cell cycle arrest or to cell death, we analyzed by FACS the cell cycle profile of RD and RH30 cell lines 72 hours after treatment with SI221 at its IC50 values. We did not find significant effects of this SFK inhibitor on cell cycle distribution (data not shown).

**Figure 2:**
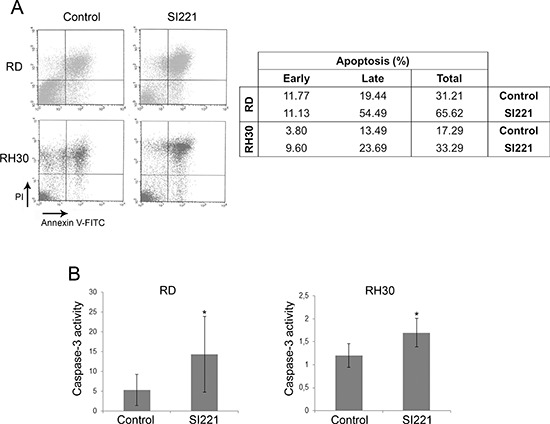
Apoptosis induction in RMS cell lines treated with SI221 **(A)** A representative FACS analysis to investigate apoptosis by cell staining with annexin V-FITC and propidium iodide (PI) of RD and RH30 cells 72 hours after treatment with SI221 or DMSO, as a control. The table reports the values relative to early apoptosis (annexin V positive and PI negative), late apoptosis (annexin V positive and PI positive) and total apoptosis. **(B)** Histograms showing caspase-3 activity in RD and RH30 cell lines 72 hours after treatment with SI221 or DMSO, as a control. Caspase-3 activity is expressed as pmol pNA/μg protein × time (hours). The reported values represent the means and standard deviations of three independent experiments. Statistically significant differences were evaluated by Student *t* test and indicated with *: significant (*P* < 0.05).

To evaluate SI221 ability to induce apoptosis in RD and RH30 cell lines, we analyzed cell staining with annexin V-FITC and PI by FACS 72 hours after treatment with SI221 at its IC50 values. Analyses of early and late apoptosis showed that SI221 was able to induce apoptosis in both the cell lines (Figure 2A). Consistently, we observed a significant increase in caspase-3 activity in RD and RH30 cells 72 hours after treatment with SI221 at its IC50 values (Figure 2B).

### SFK inhibition reduces RMS cell migration and invasion

**Figure 3:**
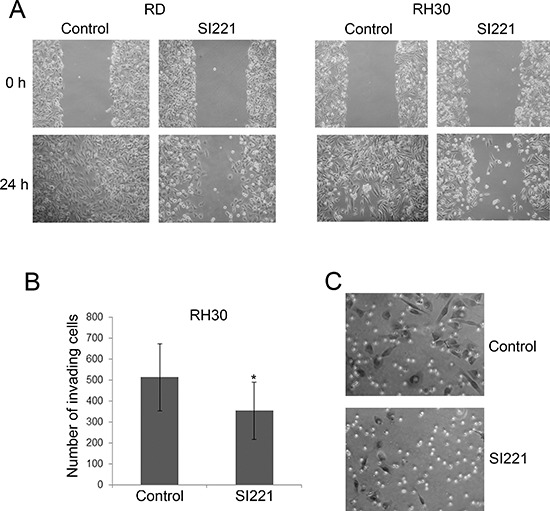
Effect of SI221 on RMS cell migration and invasion **(A)** Representative micrographs of a scratch assay conducted on RD and RH30 cells. A scratch was made in the confluent monolayer of RMS cells and photographed at 0 and 24 hours after treatment with SI221 or DMSO, as a control. Original magnification: 20X. **(B)** Histogram reporting the means and standard deviations of the number of RH30 cells that invaded through the Matrigel 24 hours after treatment with SI221 or DMSO, as a control. The number of invading cells was counted in randomly selected areas in three independent experiments. Statistically significant differences between the treated cells and the control cells were evaluated by Student *t* test and indicated with *: significant (*P* < 0.05). **(C)** Representative micrographs of RH30 cells that invaded through the Matrigel 24 hours after treatment with SI221 or DMSO, as a control. Original magnification: 40X.

In order to analyze the effect of SFK inhibition on RMS cell motility, RD and RH30 cell lines were treated with SI221 at its IC50 values (as previously calculated 72 hours after treatment) and cell migration was evaluated 24 hours after treatment by the scratch assay. We observed a sharp decrease in cell migration in both RMS cell lines (Figure 3A). In particular, in RD and RH30 control cells treated with DMSO a complete wound healing was observed, whereas in SI221 treated cell lines only a few cells migrated into the scratch. We ascertained that the number of viable cells was not significantly affected after 24 hours of SI221 treatment by trypan blue staining of RD and RH30 cells identically treated in parallel experiments (data not shown).

By using modified Boyden chambers with a Matrigel-coated filter, we also evaluated the effect of SI221 on the invasive potential of the RH30 cell line, which is representative of the most aggressive and invasive histotype [4, 14, 15]. We observed a significant decrease in cell invasion 24 hours after treatment with SI221 at its 72-hour IC50 value (Figure 3B and 3C). The number of viable cells was not significantly affected after 24 hours of treatment with SI221, as verified by trypan blue staining of RH30 cells identically treated in parallel experiments (data not shown).

### SFK inhibition induces morphological changes and myogenic marker expression in RMS cell lines

Recent data indicate that SFK inhibition is able to induce muscle differentiation in C2C12 cells [13]. Considering that RMS arises from committed skeletal muscle precursor cells that fail to differentiate and that promoting RMS differentiation is a recognized strategy to suppress the transformed phenotype [3], we set out to analyze whether SFK inhibition could be able to restore the differentiation program of RMS cells.

**Figure 4:**
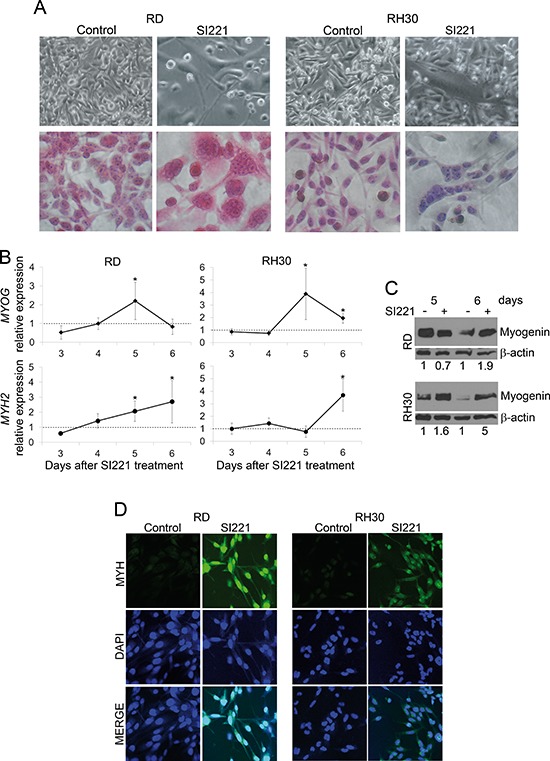
SFK inhibition-induced morphological changes and myogenic marker expression in RMS cell lines **(A)** Representative micrographs of RD and RH30 cells, both unstained (upper panel) and stained with hematoxylin and eosin (lower panel), 6 days after treatment with SI221 or DMSO, as a control. Original magnification: 40X. **(B)** Real-time qRT-PCR analysis of *MYOG* (encoding myogenin) and *MYH2* (encoding myosin heavy chain 2) expression in RD and RH30 cell lines 3, 4, 5 and 6 days after treatment with SI221. Results are reported as means of three independent experiments, each conducted in triplicate. At each evaluation time, *MYOG* and *MYH2* expression in treated cells was calculated by the 2^−ΔΔCt^ method [35] relatively to control cells, which were treated with DMSO alone and collected at the same time points. *MYOG* and *MYH2* expression values in control cells were defined as 1 and used as a baseline (dashed line). Statistically significant differences were evaluated by subjecting the ΔCt values of treated and control samples to Student *t* test, as previously indicated [38]. *: significant (*P* < 0.05). **(C)** A representative western blotting analysis of myogenin in RD and RH30 cell lines 5 and 6 days after treatment with SI221 or DMSO alone. An anti-β-actin antibody was used for a loading control. The density of the bands was quantified by densitometric analysis. Myogenin band densities were normalized with the β-actin band densities. Data are presented as relative values with respect to untreated control values, set at 1. **(D)** Representative micrographs of RD and RH30 cells treated with SI221 or DMSO, as a control, and stained by immunofluorescence with an antibody against myosin heavy chain (MYH) and by the fluorescent nuclear stain DAPI 6 days after treatment. Original magnification: 40X.

We first analyzed the morphological features of RD and RH30 cells, both unstained and stained with hematoxylin and eosin, 6 days after treatment with SI221 at its IC50 values. We observed marked changes in the morphology of the cells, which became larger, flatter and multinucleated (Figure 4A). These morphological changes seemed to be indicative of multinucleated myotube formation.

To confirm the induction of myogenic differentiation, we analyzed the expression of myogenic markers. First, we analyzed the expression of the *MYOG* gene (encoding myogenin) by real-time quantitative Reverse Transcription-PCR (qRT-PCR) after 3, 4, 5 and 6 days of treatment with SI221 at its IC50 values. We observed an increase in *MYOG* expression in both RD and RH30 cell lines 5 days after treatment (Figure 4B). This rise in *MYOG* expression decreased on day 6. Consistently, an increase in myogenin expression was also observed by western blotting 5 and/or 6 days after treatment in the two cell lines (Figure 4C).

We also analyzed the expression of the skeletal muscle *MYH2* gene (encoding myosin heavy chain 2) in RD and RH30 cell lines by real-time qRT-PCR 3, 4, 5 and 6 days after treatment with SI221 at its IC50 values. An increase in *MYH2* expression was evident 5 days after treatment and further raised after 6 days in RD cells, whereas in RH30 cells we observed an increase in *MYH2* only 6 days after treatment (Figure 4B). Consistently, by immunofluorescence analysis we also observed a sharp increase in myosin heavy chain in both RD and RH30 cells 6 days after treatment with SI221 (Figure 4D). Therefore, our data indicate that SFK inhibition is indeed able to induce RMS cell differentiation.

### SFK inhibition hinders an SFK-NOTCH3-p38 MAPK axis in RMS cell lines

In order to understand the molecular mechanisms underlying the SFK inhibition-induced RMS cell differentiation, we analyzed the effects of SFK inhibition on the expression of differentiation regulators in RMS cells.

**Figure 5:**
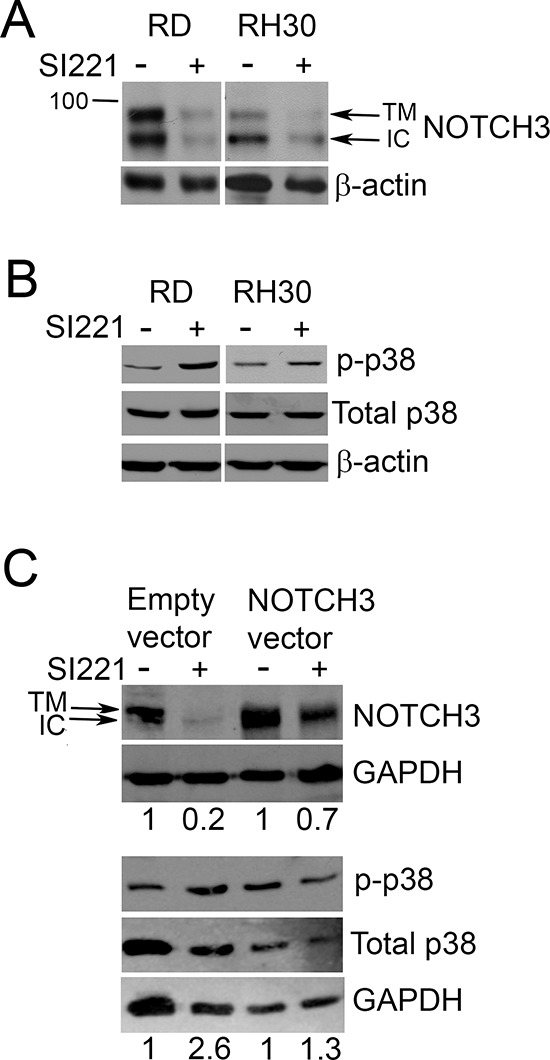
Effect of SI221 on NOTCH3 and p38 MAPK in RMS cell lines **(A)** Representative western blots of NOTCH3 in RD and RH30 cell lines treated with SI221 or DMSO alone for 72 hours. Cleaved NOTCH3 appears as a double band, with the upper band most likely representing the transmembrane (TM) form and the lower band the intracellular (IC) form. The 100 kDa band of a molecular weight marker is shown. An equal loading of proteins was verified with an anti-β-actin antibody. **(B)** A representative western blotting analysis of the active phosphorylated form of p38 MAPK (p-p38) and total level of p38 MAPK in RD and RH30 cell lines treated with SI221 or DMSO alone for 72 hours. An anti-β-actin antibody was used for a loading control. **(C)** Representative western blots of cleaved NOTCH3, phospho-p38 MAPK (p-p38) and total p38 MAPK in RH30 cells transfected with a vector expressing the constitutively active intracellular form of NOTCH3 or with a control empty vector and treated with SI221 or DMSO alone for 72 hours. An anti-GAPDH antibody was used for a loading control. The density of the bands was quantified by densitometric analysis. NOTCH3 band densities were normalized with the GAPDH band densities, whereas p-p38 band densities were normalized with those of total p38. Data are presented as relative values with respect to untreated control values, set at 1.

Recent data implicated SFKs in the NOTCH pathway, showing that SFK inhibition decreased the active cleaved form of NOTCH1 in pancreatic cancer cells [16]. This is particularly intriguing because NOTCH signaling is involved in RMS development. Indeed, NOTCH signaling is upregulated in RMS and its inhibition reduces RMS cell proliferation and invasion and promotes differentiation [15, 17–19]. In particular, among NOTCH family members, NOTCH1 seems to play a role in hindering differentiation in RD cells but not in RH30 cells, NOTCH3 has a role in inhibiting differentiation in both RD and RH30 cells, whereas NOTCH2 seems not to be involved in differentiation inhibition in RMS cells and NOTCH4 is undetectable in these cells [19]. Therefore, we investigated the effect of SI221 on NOTCH3 expression and found that SI221, at its IC50 values, decreased the cleaved form of NOTCH3 in both RD and RH30 cell lines 72 hours after treatment (Figure 5A).

Recent findings also show that SFK inhibition is able to induce muscle differentiation in C2C12 cells through p38 MAPK activation [13]. Since p38 MAPK is impaired in RMS cells and its activation can induce RMS differentiation [20], we analyzed the effect of SI221 on p38 MAPK activation in RMS cell lines. As expected, we observed an increase in the active phosphorylated form of p38 MAPK in both RMS cell lines 72 hours after treatment with SI221 at its IC50 values (Figure 5B).

These data suggest that SI221 likely triggers the recovery of the differentiation program in RMS cells by decreasing cleaved NOTCH3, which inhibits muscle differentiation, and increasing the active form of p38 MAPK, which promotes differentiation.

A possible functional link between the SFK inhibition-induced NOTCH3 downregulation and p38 MAPK activation observed in RMS cells is provided by a study reporting that NOTCH signaling is able to suppress the activity of p38 MAPK in C2C12 cells [21]. Consistently, *NOTCH3* inhibition by RNA interference activates p38 MAPK in RMS cell lines [19]. Therefore, in order to evaluate whether the observed SI221-induced increase in phospho-p38 MAPK was dependent on SI221 ability to decrease NOTCH3, we transiently transfected the RH30 cells to induce mild levels of the constitutively active intracellular form of NOTCH3 and analyzed the expression of phospho-p38 MAPK after 72 hours of treatment with SI221. As a consequence of SI221 inability to inhibit the constitutively active form of NOTCH3, SI221 was no longer able to increase significantly phospho-p38 MAPK levels (Figure 5C). However, although SI221 was not able to inhibit the transfected constitutively active form of NOTCH3, the compound was still able to inhibit the endogenous NOTCH3, thus explaining the observed slight decrease in total NOTCH3 after SI221 treatment. As expected, in cells transfected with a control empty vector, SI221 was confirmed to be able to strongly inhibit NOTCH3 and increase markedly phospho-p38 MAPK. Therefore, our data indicate that SI221 ability to trigger p38 MAPK activation was a consequence of its inhibitory action on NOTCH3.

### Pharmacological inactivation of p38 MAPK prevents the SFK inhibitor-induced RMS cell differentiation

**Figure 6:**
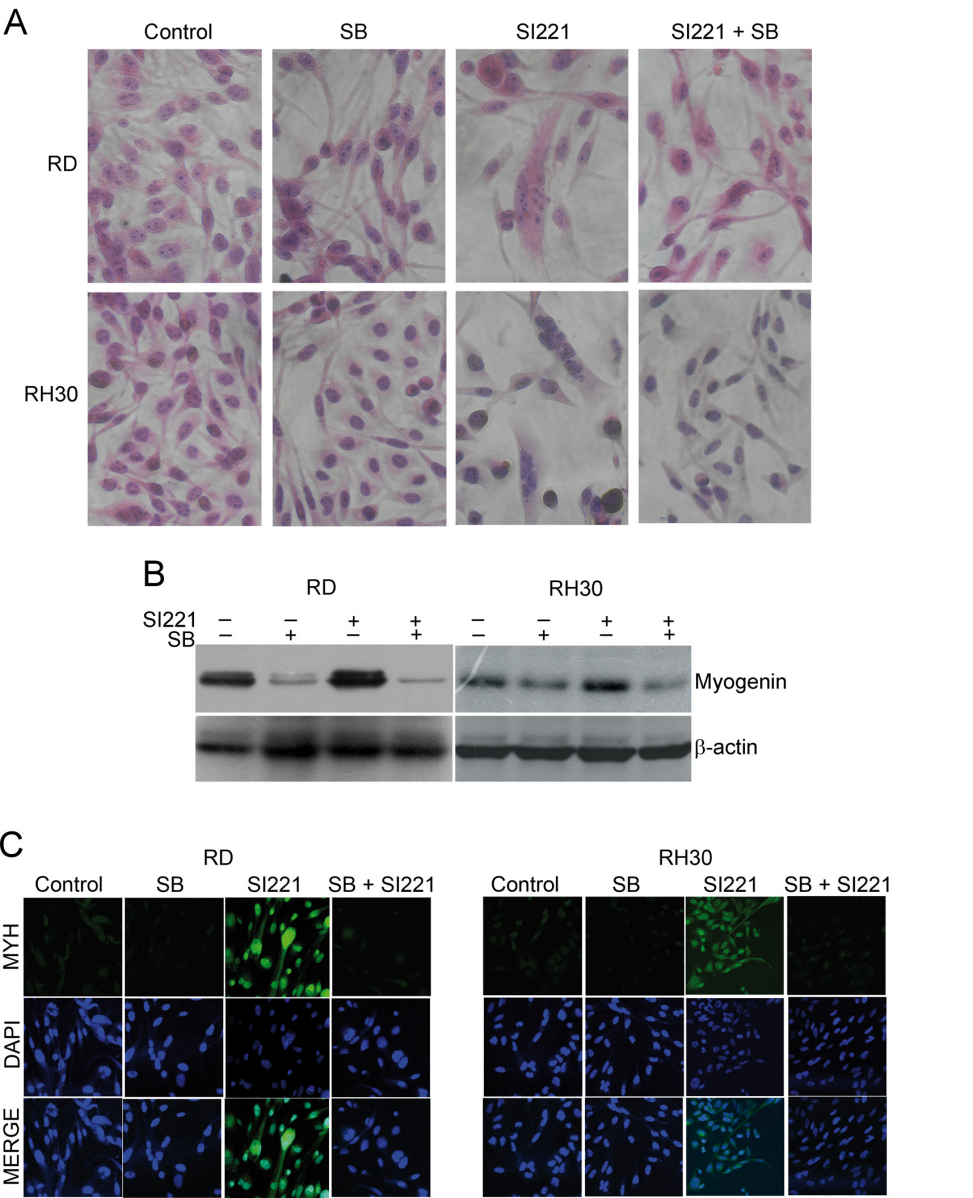
Effect of p38 MAPK inactivation on SFK inhibitor-induced RMS cell differentiation **(A)** Representative micrographs of RD and RH30 cells stained with hematoxylin and eosin 6 days after treatment with the p38 MAPK inhibitor SB203580 and/or SI221 or DMSO, as a control. Original magnification: 40X. **(B)** A representative western blotting analysis of myogenin in RD and RH30 cell lines 6 days after treatment with SB203580 and/or SI221 or DMSO. An anti-β-actin antibody was used for a loading control. **(C)** Representative micrographs of RD and RH30 cells treated with SB203580 and/or SI221 or DMSO and analysed by immunofluorescence upon staining with an antibody against myosin heavy chain (MYH) and the fluorescent nuclear stain DAPI 6 days after treatment. Original magnification: 40X.

To verify whether p38 MAPK activation is indeed required for the differentiation program triggered by SFK inhibition in RMS cells, we treated RD and RH30 cell lines with 10 μM SB203580, a well-known p38 MAPK inhibitor, both alone and in combination with SI221 at its IC50 values for 6 days. As expected, p38 MAPK inactivation inhibited the SI221-induced muscle differentiation, as shown by both cell morphology (Figure 6A) and myogenin and myosin expression (Figure 6B and 6C).

### SI221 reduces tumor growth in a xenograft model of RMS

**Figure 7:**
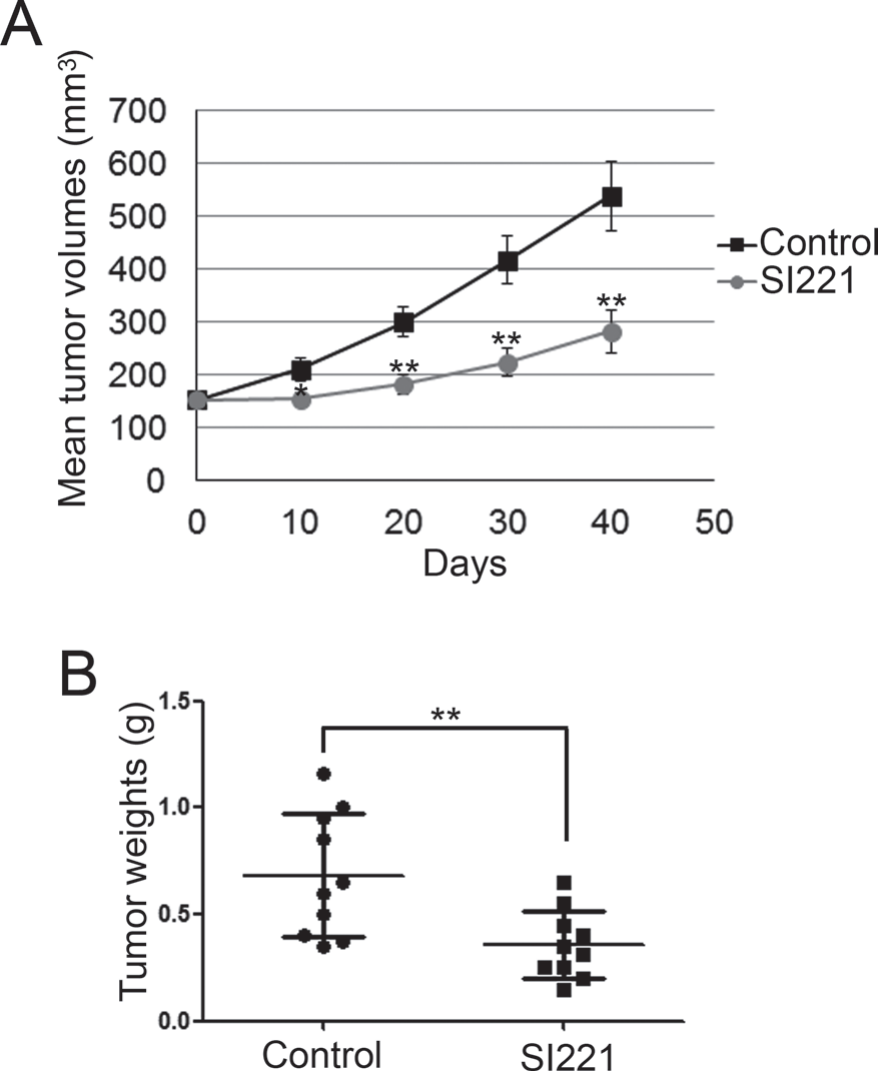
SI221 reduces RMS cell growth in xenotrasplanted mice **(A)** Growth curves of RD xenografts in 2 groups of nude mice treated with vehicle alone (control) or SI221, respectively. The reported values represent the means and standard errors of the tumor volumes at each measurement. Statistically significant differences between the mean tumor volumes of control mice and SI221-treated mice were evaluated by Student *t* test at each evaluation time and indicated with *: significant (*P* < 0.05) and **: very significant (*P* < 0.01). **(B)** Dot plots showing the means and standard deviations of the weights of tumors excised from control mice and SI221-treated mice at the end of treatment. Statistical significance was evaluated through Student *t* test and indicated with**: very significant (*P* < 0.01).

We also analyzed the ability of SI221 to reduce tumor growth in a pilot *in vivo* experiment. Two groups of nude mice (10 each) carrying RD xenografts received 50 mg/kg of SI221 or vehicle only, respectively, three times a week *per os*. Tumor volumes were measured 10, 20, 30 and 40 days after treatment. Our results showed that SI221 treatment significantly reduced the growth rate of RD xenografts (Figure 7A). Consistently, analysis of tumor weights excised from mice at the end of the study period showed that the mean tumor weight was significantly lower in SI221-treated mice than in control mice (Figure 7B).

## DISCUSSION

Over the last decades, many efforts have been devoted to improve therapeutic options for RMS, leading to the development of a multimodality therapy, consisting of systemic chemotherapy in combination with surgery and radiation therapy, which has determined a significant improvement in survival [3]. Nevertheless, patients with metastatic disease and high risk features still face a poor prognosis and, considering that RMS primarily affects young patients, there are concerns about long-term adverse effects of the treatment [1, 3]. Therefore, the development of more selective targeted therapies against RMS is an urgent need.

It has recently been shown that SFK hyperactivation could play a role in RMS development [7, 8]. Inhibition of SFKs, whose aberrant activation is implicated in the onset and progression of many human cancers, is considered one of the most promising strategies for cancer therapy [6]. In RMS cell lines, dasatinib-mediated SFK inhibition induced an antiproliferative effect both *in vivo* and *in vitro* [8] and a reduction in cell migration [7], thus suggesting that SFKs could represent therapeutic targets also for RMS.

In the present study, we challenged two RMS cell lines (the RD and RH30 cells, representing the ERMS and ARMS subtypes, respectively) with new pyrazolo[3,4-*d*]pyrimidine derivative SFK inhibitors, whose efficacy was previously demonstrated in several tumor types [9–12]. Our results showed that these SFK inhibitors exerted a significant cytotoxic effect on these cell lines. We focused on one of these SFK inhibitors, the SI221 compound, which was able to reduce cell viability in both the RMS cell lines, without significantly affecting non-tumor cells (primary human skin fibroblasts and differentiated C2C12 cells).

We verified that SI221 effectiveness was indeed dependent on SFK activation status. SI221 proved to be effective on RMS cells expressing the active phosphorylated form of SFKs and, as expected, caused a strong decrease in phospho-SFK levels, whereas it was ineffective on fibroblasts, in which the active form of SFKs was almost undetectable. Further confirming the specificity of SI221 towards active SFKs, SI211 effectiveness decreased during C2C12 differentiation, mirroring the reduction in the expression of the active form of SFKs.

We also tested the selectivity of SI221 in a kinase activity screening assay and found that this compound specifically inhibited SFKs, without affecting the activity of the other kinases included in the panel. Moreover, SI221 proved to be mainly effective against the SFK member YES, which has recently been recognized as a player in RMS development, as shown by the high expression of *YES* mRNA in RMSs compared with normal tissues, YES hyperactivation in RMS cell lines, and the ability of short hairpin RNAs against *YES* to inhibit RMS cell growth [8].

To date, several small molecules targeting SFKs have been developed [9]. In the present study, we compared the effectiveness of SI221 with that of the well-known SFK inhibitor PP2. Consistent with our previous works showing that PP2 was less effective in different tumor types compared with our compounds [11, 12, 22–24], we observed that PP2 was less effective than SI221 in the RD cell line. However, in RH30 cells the two inhibitors showed a similar efficacy.

We then evaluated whether the cytotoxic effects induced by SFK inhibition on RMS cells were due to cell cycle arrest or to cell death. We did not find significant effects on cell cycle distribution 72 hours after treatment. Conversely, consistent with the ability of various SFKs to activate antiapoptotic pathways [25, 26], we observed that SFK inhibition through SI221 induced apoptosis in RMS cell lines.

It is well-established that the most studied SFK member, SRC, is also involved in promoting cell motility and invasion. Indeed, increased SRC kinase activity is associated with the metastatic potential of many tumor types and SRC has an important role in regulating focal adhesion and adherent junctions, which are the principal subcellular structures involved in cell adhesion, motility and invasion [6]. Therefore, we analyzed the effect of SI221 on the ability of RMS cells to migrate and invade and found a decrease in both these processes after treatment with SI221.

Considering that RMS arise from committed skeletal muscle precursor cells that fail to differentiate and that the induction of RMS differentiation is a recognized strategy to suppress the transformed phenotype [3], we analyzed whether SFK inhibition could be able to restore the differentiation program in RMS cells. We observed evident morphological changes in SI221-treated RD and RH30 cells, which became larger, flatter and multinucleated. These morphological changes seemed indicative of multinucleated myotube formation, although nuclei were not arranged linearly as in myotubes. Similar morphological changes were previously observed in RMS cell lines and demonstrated to be associated with myogenic differentiation [27]. Consistently, we observed an increase in myogenic markers (myogenin and MYH) in RMS cells treated with SI221.

MYH was expressed both in the nuclei and cytoplasm of SI221-treated RMS cells, consistent with what previously observed in RMS cells that underwent differentiation in response to different stimuli [19, 20]. This diffuse pattern of MYH expression could seem inconsistent with the typical cytoplasmic localization of myosin. However, although myosin was classically thought to function only in the cytoplasm, it is now well-established that different myosin types also have specific nuclear roles [28]. In particular, embryonic myosin has also been found in nuclei [28] and this myosin type represents a differentiation marker for RMS [29, 30]. Therefore, considering that the antibody (MF-20) used in this study recognizes MYH at all stages of embryonic development [31], it is likely that the MYH found in nuclei of differentiating RMS cells under SI221 treatment is the embryonic isoform, although this needs to be further assessed.

To dissect the molecular mechanisms underlying SFK inhibition-induced RMS cell differentiation, we analyzed whether SFK inhibition could be able to modulate the expression of differentiation regulators in RMS cells. Given the recently recognized role of SFKs in NOTCH [16] and p38 MAPK [13] pathways, we focused on the effects of SFK inhibition on the expression of these proteins, which play opposite roles in muscle differentiation and whose expression is altered in RMS. In particular, NOTCH signaling has a role in inhibiting muscle differentiation and is upregulated in RMS cells [15, 17–19], whereas p38 MAPK promotes muscle differentiation [32] and is impaired in RMS cells [20].

Consistent with the recent observation that SFK inhibition was able to decrease the active cleaved form of NOTCH1 in pancreatic cancer cells [16], we found that SI221 greatly decreased the cleaved form of NOTCH3 in both RD and RH30 cell lines. This is noteworthy because *NOTCH3* inhibition by RNA interference was recently shown to induce myogenic differentiation in both RD and RH30 cells [19].

Moreover, we observed that SI221 activated p38 MAPK in both RMS cell lines and that the pharmacological inactivation of p38 MAPK inhibited the SI221-induced muscle differentiation, thus demonstrating that p38 MAPK is required for the differentiation program triggered by SFK inhibition in RMS cells. This finding is consistent with previous studies showing that SFK inhibition is able to induce muscle differentiation in C2C12 cells through p38 MAPK activation [13] and that p38 MAPK constitutive activation is able to induce RMS differentiation [20].

Therefore, our data indicate that SFK inhibition can trigger the recovery of the differentiation program in RMS cells by decreasing cleaved NOTCH3, which inhibit muscle differentiation, and increasing the active form of p38 MAPK, which promotes differentiation.

We also showed, through the transfection of the constitutively active intracellular form of NOTCH3, that the ability of SI221 to induce an increase in phospho-p38 MAPK was a secondary consequence of its inhibitory action on NOTCH3. This observation is consistent with previous studies reporting that NOTCH signaling is able to suppress the activity of p38 MAPK in C2C12 cells [21] and that *NOTCH3* inhibition by RNA interference activates p38 MAPK in RMS cell lines [19]. Overall, our findings indicate that inhibiting SFKs could induce the differentiation program in RMS cells by hindering an SFK–NOTCH3–p38 MAPK axis.

We also observed that SI221 was able to reduce tumor growth in a xenograft model of RMS. Thus, although further experiments are required to assess whether SI221 acts *in vivo* through the same mechanisms observed *in vitro*, this pilot *in vivo* study suggests that specific SFK inhibition could represent a feasible approach for RMS treatment.

In conclusion, we observed that SFK inhibition through SI221 reduced RMS cell viability and migration *in vitro* and decreased tumor growth in xenotrasplanted mice. Our data also strongly suggest that SFK inhibition can induce RMS differentiation by hindering an SFK-NOTCH3–p38 MAPK axis. Thus, our observations define a possible new mechanism involving SFKs in RMS cell failure to differentiate and provide a new rationale for targeting SFKs in RMS. Indeed, SFK inhibition, besides reducing tumor cell growth and invasive potential, could also represent a differentiation therapeutic strategy for RMS.

## METHODS

### Cell culture

RD and RH30 rhabdomyosarcoma cell lines were recently purchased from American Type Culture Collection (ATCC) and Leibniz Institute DSMZ-German Collection of Microorganisms and Cell Cultures, respectively, and maintained at a low passage number. The mouse myoblast cell line C2C12 was purchased from ATCC and primary human skin fibroblasts were a kind gift of Michele Fimiani, Giancarlo Mariotti and Stefania Mei (University of Siena, Italy) [33].

Cells were maintained in DMEM (RD, fibroblasts and C2C12) and RPMI-1640 (RH30) containing 10% fetal bovine serum (FBS) and 2 mM L-Glutamine.

For differentiation induction, C2C12 cells were cultured until subconfluence in growth medium (GM), as described above, and then shifted to DM consisting of DMEM containing 2% horse serum.

### Cell treatment with the SFK inhibitors, MTS assay and kinase activity screening assay

The pyrazolo[3,4-*d*]pyrimidine derivative SFK inhibitor SI221 was synthesized as previously reported [34]. SI221 and the commercially available SFK inhibitor PP2 (4-amino-5-(4-chlorophenyl)-7-(dimethylethyl)pyrazolo[3,4-*d*]pyrimidine, Calbiochem) were dissolved in DMSO (Sigma-Aldrich) to a concentration of 20 mM and then diluted in culture medium before use.

RD, RH30, C2C12 cell lines and fibroblasts were seeded in 96-well plates 24 hours before treatment with the SFK inhibitors at concentrations ranging from 1 to 15 μM. Control cells were treated with DMSO at the maximum amount used to deliver the SFK inhibitors. DMSO showed no toxic effect on all the cell lines (data not shown). Seventy-two hours after treatment, cell viability was evaluated by MTS assay (CellTiter 96® AQueous One Solution Cell Proliferation Assay, Promega), following the manufacturer's instructions, through spectrophotometric reading at two different wavelengths (540 and 630 nm). IC50 values were calculated using GraphPad Prism Software, version 5.01 for Windows. To calculate the IC50 of C2C12 cells, the SFK inhibitor concentration range was extended up to 25 μM.

To evaluate the selectivity of SI221 towards the SFK members, we tested this SFK inhibitor, at the concentration of 10 μM, against a panel of 29 different kinases, including 8 SFKs (Merck Millipore KinaseProfiler service).

### Protein extraction and western blotting

Cells were harvested on ice in lysis buffer consisting of 50 mM Tris-HCl pH 7.5, 50 mM EDTA pH 8, 150 mM NaCl, 1% NP40, 2 mM Na_3_VO_4_, 10 mM NaF, 0.3 mM PMSF, a protease inhibitor cocktail (Roche) and the phosphatase inhibitor cocktail 3 (Sigma-Aldrich). Equal amounts of proteins (50 μg) per sample were electrophoresed and blotted onto PVDF membranes (Bio-Rad), which were then blocked in 5% nonfat dry milk or 5% BSA (for antibodies against phospho-proteins) and incubated with antibodies against: phospho-SRC family (Tyr416) (this antibody detects SRC only when phosphorylated at Tyr416, which corresponds to human Tyr419. It cross-reacts with the other SFK members when phosphorylated at equivalent sites), SRC, FYN, LCK, LYN, YES, phospho-p38 MAPK (Thr180/Tyr182), p38 MAPK (Cell Signaling); BLK, FGR, HCK, MYH, myogenin, NOTCH3, GAPDH (Santa Cruz Biotechnology); and β-actin (Sigma-Aldrich). After incubation with horseradish peroxidase-conjugated secondary antibodies, signals were detected using the Supersignal West Pico Chemiluminescent Substrate (Pierce) and autoradiography films (Pierce).

### Cytoflourimetric analyses of cell cycle profile and apoptosis

RD and RH30 cells were seeded in 100 mm diameter petri dishes and, 24 hours after seeding, treated with SI221 at its IC50 values or DMSO. Seventy-two hours after treatment, cells were harvested, washed with PBS and fixed in 70% ice-cold ethanol. Nuclei were stained with 5 μg/ml propidium iodide (PI) plus 20 μg/ml RNase (Sigma-Aldrich) at 4°C overnight in the dark and analyzed with a BD FACSCanto II flow cytometer (Becton Dickinson). Data were analyzed using a BD FACSDiva software (Becton Dickinson).

Apoptosis was evaluated by cell staining with annexin V-FITC and PI (Annexin V-FITC kit, Miltenyi Biotec Inc.) and FACS analysis 72 hours after treatment, according to the manufacturer's instructions.

### Caspase-3 activity assay

RD and RH30 cells were seeded and treated for 72 hours with SI221 or DMSO, as described above. Caspase-3 enzymatic activity was evaluated in cellular lysates using the Colorimetric Caspase-3 Assay Kit (Sigma-Aldrich) following the manufacturer's instructions. This assay is based on the hydrolysis of the peptide substrate acetyl-Asp-Glu-Val-Asp p-nitroanilide by caspase-3, resulting in the release of the p-nitroaniline (pNA) moiety. The absorbance of pNA was measured by spectrophotometry at 405 nm through a microplate reader. pNA pmols were determined by a calibration curve prepared with defined pNA solutions. Caspase-3 activity is expressed as pmol pNA/μg protein × time (hours).

### Migration and invasion analysis

To analyze the effect of SFK inhibition on RMS cell motility, we performed scratch assays. In particular, RD and RH30 cells were grown to confluence on tissue culture plates and a scratch was made in the confluent monolayer using a sterile pipette tip. Cells were washed with PBS and then complete medium containing either SI221 at its IC50 values (as previously evaluated at 72 hours after treatment) or DMSO, as a control, was added. Cells were photographed at 0 and 24 hours after treatment.

The effect of SI221 on the invasive ability of RH30 cells was evaluated using 6-well modified Boyden chambers with Matrigel-coated 8 μm pore size membranes (Becton Dickinson). Cells were placed in the upper compartment in medium containing 3% FBS, whereas serum-free medium was placed in the lower compartment. Cells were allowed to adhere for 16 hours and then treated with SI221 at its 72-hour IC50 value or with DMSO, as a control. Medium containing 20% FBS was placed in the bottom compartment, as a chemoattractant. Twenty-four hours after treatment cells on top of the membrane were scraped off and discarded. Cells that invaded through the Matrigel were fixed and stained with hematoxylin and eosin. After removing the membrane with a scalpel blade and placing it on a microscope slide, cells were counted by observation through light microscopy. Twenty fields chosen randomly from both the center and periphery of the membrane were examined and three separate experiments were conducted.

To verify that the number of viable cells was not significantly affected by SI221 treatment at the culture times used for the migration and invasion assays, we also performed parallel experiments in which cells were identically treated in 6-well tissue culture plates and then harvested and assessed for cell viability by trypan blue staining.

### Real-time quantitative reverse transcription-PCR

Total RNA was extracted from RD and RH30 cells, treated for 3, 4, 5 and 6 days with either SI221 (at its IC50 values) or DMSO, using the miRNeasy MiniKit (Qiagen) according to the manufacturer's instructions. The expression of *MYOG* and *MYH2* was analyzed by real-time qRT-PCR. Five hundred nanograms of RNA were reverse transcribed with the iScript cDNA Synthesys kit (Bio-Rad). One microliter of cDNA was amplified in the Opticon 2 real-time PCR cycler (MJ Research) using the Sso Fast EvaGreen Supermix (Bio-Rad) following the manufacturer's instructions. Forty cycles of PCR were performed using an annealing temperature of 60°C. The mRNA levels were normalized to those of the housekeeping *GAPDH* gene. *MYOG* and *MYH2* expression in treated cells was calculated by the 2^−ΔΔCt^ method [35] relatively to control cells. Primer sequences were 5′-CAGCGAATGCAGCTCTC ACA-3′ and 5′-AGTTGGGCATGGTTTCATCTG-3′ for *MYOG* [36]; 5′-CTGATGCCATGGAATGACTG-3′ and 5′-CCCTATGCTTTATTTCCTTTGC-3′ for *MYH2* [37] and 5′-AGCCCCCGGACCTGCACT-3′ and 5′-CCGG CAAAAACAAATAAGTTGACT-3′ for *GAPDH*.

### Immunofluorescence

RD and RH30 cells were grown on cover slips and treated with SI221 at its 72-hour IC50 values or DMSO, as a control. Six days after treatment, cells were fixed in 4% paraformaldehyde for 15 min and permeabilized by 0.2% Triton X-100 in PBS for 10 min. Samples were then blocked in 1% BSA for 20 min and then incubated with the MF-20 antibody (Developmental Studies Hybridoma Bank at the University of Iowa) for 2 h. After washing, samples were incubated with Alexa Fluor® 488 Goat Anti-Mouse IgG (H+L) Antibody (Life Technologies) at 37°C for 45 min. The coverslips were mounted using the ProLong Gold Antifade Reagent with DAPI (Life Technologies). Images were obtained using a Zeiss Axiovert 100 M confocal microscope.

### Cell transfection

RH30 cells were seeded in 100 mm diameter petri dishes and, 24 hours after seeding, transfected with 10 μg of a vector expressing the intracellular form of NOTCH3 (Addgene) or a control empty vector through the TransIT®-2020 tranfection reagent (Mirus Bio LLC), according to the manufacturer's instructions. Upon transfection, cells were treated with either SI221 (at its IC50 values) or DMSO, as a control, and total proteins were extracted after 72 hours for western blotting analysis.

### Cell treatment with the p38 MAPK inhibitor SB203580

RD and RH30 cells were seeded in 100 mm diameter petri dishes and on cover slips. Twenty-four hours after seeding, cells were treated with 10 μM SB203580 (Calbiochem) and/or SI221 at its 72-hour IC50 values, or DMSO. Six days after treatment, cells were fixed and stained with hematoxylin and eosin or by immunofluorescence or harvested for western blotting analysis.

### Xenograft experiments

All experimental procedures involving animals and their care were approved by the Institutional Review Board and ethic committee of the University of L'Aquila in compliance with national and international laws and policies. RD cells were cultured in standard conditions until reaching the 80% of confluence, then were detached with a trypsin/EDTA solution, counted and adjusted at a density of 2 × 10^6^ cells per 200 μl of 10 mg/ml Matrigel solution in saline buffer. Cancer cells were injected subcutaneously into the flank of immunodeficient mice (CD1 nude mice from Charles River Laboratories International, Inc.). Before any invasive manipulation, mice were anesthetized with a mixture of ketamine (25 mg/ml)/xylazine (5 mg/ml). Tumor growth was monitored daily and measured by caliper. When tumor mass was evident (100–200 mm^3^) mice were assigned randomly to different experimental groups: a control group receiving the vehicle (0.5% methylcellulose) and a treatment group receiving 50 mg/kg of SI221 three times a week *per os*. After 40 days tumors were harvested and weighed.

### Statistical analysis

Statistical analyses were performed using the GraphPad Prism Software, version 5.01 for Windows. Statistically significant differences among the means of multiple matched groups were evaluated by one-way repeated measures Anova with Dunnett post-test, to compare all data vs control (MTS assay). To evaluate statistically significant differences between the means of two matched groups, as for invasion analysis, caspase-3 assay and real-time qRT-PCR [38], we used paired Student *t* test. The unpaired Student *t* test was used to compare the mean tumor volumes between control and SI221-treated xenotransplanted mice at each evaluation time. The unpaired Student *t* test was also used to compare the mean weights of tumors excised from control and SI221-treated mice at the end of treatment. *P* < 0.05 was considered to be statistically significant.

## SUPPLEMENTARY FIGURE AND TABLE



## Abbreviations

ARMS: alveolar rhabdomyosarcoma
DM: differentiation medium
ERMS: embryonal rhabdomyosarcoma
FBS: fetal bovine serum
GM: growth medium
IC: intracellular
IC50: half maximal inhibitory concentration
MYH: myosin heavy chain
MYOG: myogenin
p38 MAPK: p38 mitogen activated protein kinase
PI: propidium iodide
pNA: p-nitroaniline
qRT-PCR: quantitative Reverse Transcription-PCR
RMS: rhabdomyosarcoma
SFKs: SRC family kinases
TM: transmembrane
