# Supplementary material for: SRC family kinase (SFK) inhibition reduces rhabdomyosarcoma cell growth in vitro and in vivo and triggers p38 MAP kinase-mediated differentiation
Source: Oncotarget. 2015 Feb 3;6(14):12421–35. doi: 10.18632/oncotarget.3043 (PMC4494948; doi:10.18632/oncotarget.3043)
Supplement: Supplementary file 1 [file oncotarget-06-12421-s001.pdf]

SUPPLEMENTARY FIGURE AND TABLE

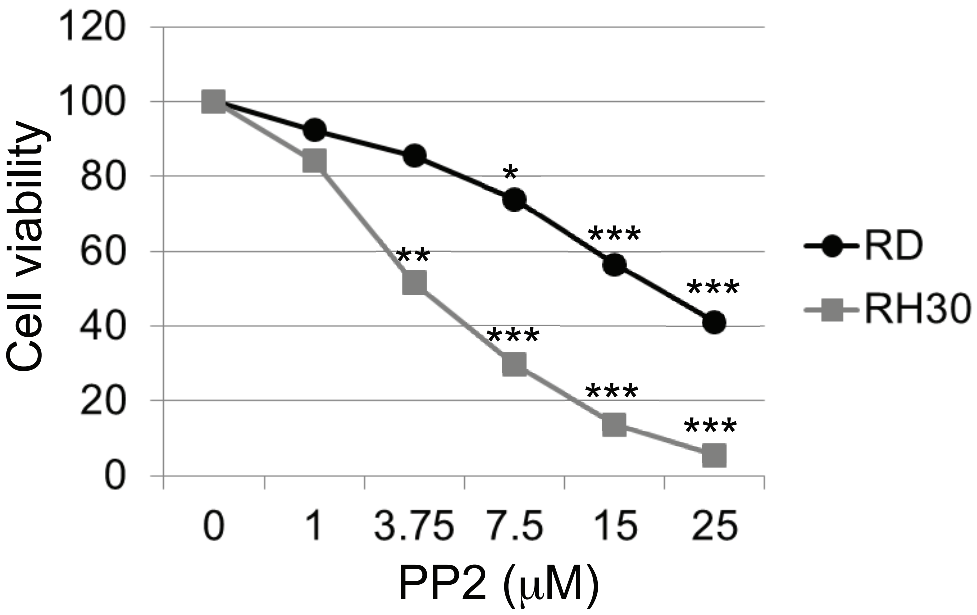

IC50 values (μM)	
Cell lines	PP2
RD	19.12
RH30	3.79

Supplementary Figure 1: MTS analysis of cell viability in RMS cell lines (RD and RH30) 72 hours after treatment with PP2. Results are reported as means of three independent experiments, each conducted in triplicate, and expressed as percentages of cell viability calculated with respect to control cells treated with DMSO alone. The absorbance values of treated and control samples were subjected to one-way Anova with Dunnett post-test. Statistically significant differences between treated and control cells are indicated with *: significant ($P < 0.05$), **: very significant ($P < 0.01$) and ***: extremely significant ($P < 0.001$). The table reports the IC50 values of PP2 on RMS cell lines.

Supplementary Table S1: Kinase selectivity profile of SI221 (10 µM)

Kinase	Residual activity (%)
BLK	75
CHK1	99
cKIT	97
CSK	92
EGFR	96
EPHA2	101
FAK	102
FGFR1	83
FGFR2	91
FGFR3	95
FGR	70
FYN	71
GSK3β	94
HCK	28
IGF-1R	98
JAK2	107
LCK	53
LYN	66
MET	75
mTOR	95
PDGFRα	96
PDGFRβ	94
PIM-1	90
PKA	100
PKCα	106
ROCK-I	93
SRC	65
SYK	112
YES	23

Shading indicates SFK members
